# Defibrotide inhibits donor leucocyte‐endothelial interactions and protects against acute graft‐versus‐host disease

**DOI:** 10.1111/jcmm.15434

**Published:** 2020-06-10

**Authors:** David García‐Bernal, Marta Palomo, Carlos M. Martínez, José E. Millán‐Rivero, Ana I. García‐Guillén, Miguel Blanquer, Maribel Díaz‐Ricart, Robert Sackstein, Enric Carreras, Jose M. Moraleda

**Affiliations:** ^1^ Hematopoietic Transplant and Cellular Therapy Unit Instituto Murciano de Investigación Biosanitaria IMIB‐Arrixaca Virgen de la Arrixaca University Hospital University of Murcia Murcia Spain; ^2^ Internal Medicine Department, Medicine School University of Murcia Murcia Spain; ^3^ Josep Carreras Leukaemia Research Institute Barcelona Spain; ^4^ Hematopathology Department of Pathology Centre de Diagnostic Biomedic (CBD), Hospital Clinic de Barcelona Institut d’Investigacions Biomediques August Pi i Sunyer (IDIBAPS) Universitat de Barcelona Barcelona Spain; ^5^ Barcelona Endothelium Team Barcelona Spain; ^6^ Experimental Pathology Unit Instituto Murciano de Investigación Biosanitaria IMIB‐Arrixaca Murcia Spain; ^7^ Department of Translational Medicine, and the Translational Glycobiology Institute Herbert Wertheim College of Medicine Florida International University Miami FL USA

**Keywords:** acute GvHD, defibrotide, hematopoietic stem cell transplantation

## Abstract

Allogeneic hematopoietic stem cell transplantation (allo‐HCT) is an effective therapy for the treatment of high‐risk haematological malignant disorders and other life‐threatening haematological and genetic diseases. Acute graft‐versus‐host disease (aGvHD) remains the most frequent cause of non‐relapse mortality following allo‐HCT and limits its extensive clinical application. Current pharmacologic agents used for prophylaxis and treatment of aGvHD are not uniformly successful and have serious secondary side effects. Therefore, more effective and safe prophylaxis and therapy for aGvHD are an unmet clinical need. Defibrotide is a multi‐target drug successfully employed for prophylaxis and treatment of veno‐occlusive disease/sinusoidal obstruction syndrome. Recent preliminary clinical data have suggested some efficacy of defibrotide in the prevention of aGvHD after allo‐HCT. Using a fully MHC‐mismatched murine model of allo‐HCT, we report here that defibrotide, either in prophylaxis or treatment, is effective in preventing T cell and neutrophil infiltration and aGvHD‐associated tissue injury, thus reducing aGvHD incidence and severity, with significantly improved survival after allo‐HCT. Moreover, we performed in vitro mechanistic studies using human cells revealing that defibrotide inhibits leucocyte‐endothelial interactions by down‐regulating expression of key endothelial adhesion molecules involved in leucocyte trafficking. Together, these findings provide evidence that defibrotide may represent an effective and safe clinical alternative for both prophylaxis and treatment of aGvHD after allo‐HCT, paving the way for new therapeutic approaches.

## 1 INTRODUCTION

Acute graft‐versus‐host disease (aGvHD) is the most frequent life‐threatening complication after allogeneic hematopoietic stem cell transplantation (allo‐HCT). The pathobiology of aGVHD results from immunocompetent allogeneic T cells contained in the marrow graft that recognize the recipient as foreign and migrate and attack specific host tissues (typically the skin, gastrointestinal tract and liver), resulting in significant immune/cytokine‐mediated tissue injury, endothelial dysfunction, and high morbidity and mortality.^1^, ^2^, ^3^, ^4^, ^5^ Different prophylactic and treatment regimens are currently employed to reduce the incidence and severity of aGvHD, most commonly based on corticosteroids, calcineurin inhibitors or other immunosuppressants. However, responses are unsatisfactory and aGvHD remains a significant problem. Thus, there is an unmet need for the development of newer strategies to prevent and treat this disease.^6^

Defibrotide is a polydisperse mixture of 90% single‐stranded and 10% double‐stranded phosphodiester oligonucleotides derived from controlled depolymerization of porcine intestinal mucosal DNA that has proved to be effective for prophylaxis and treatment of hepatic veno‐occlusive disease/sinusoidal obstruction syndrome (VOD/SOS) following allo‐HCT.^7^, ^8^ It has been proposed to have a protective effect on activated endothelial cells,^9^, ^10^, ^11^ and among others, pro‐fibrinolytic, anti‐thrombotic and anti‐inflammatory activities have been reported.^12^ Defibrotide has been found to suppress heparanase gene expression, whose high levels have been postulated as a risk factor for aGvHD development,^13^, ^14^ and it was initially identified as an adenosine receptor agonist, whose activation inhibits T cell function.^10^, ^15^, ^16^ Importantly, preliminary evidence in recent clinical trials employing defibrotide for VOD suggests that it could also have a prophylactic effect on the occurrence of aGvHD after allo‐HCT: patients who received VOD prophylaxis with defibrotide had also a lower incidence and severity of aGvHD than untreated control group of patients, and it also did not seem to interfere with a graft‐versus‐leukaemia effect.^17^, ^18^ Based on these indirect data, defibrotide received an orphan designation for the prevention of aGvHD. However, its efficacy for the prevention and treatment of aGvHD as the only drug without the administration of other immunosuppressant/s, as well as the molecular mechanisms mediating its aGvHD‐protective effect, has not been previously addressed.

Given such prior findings, we sought the mechanistic basis of defibrotide effects on evolving aGvHD. Here, using a murine model of fully major histocompatibility complex (MHC)‐mismatched allo‐HCT to induce fulminant aGvHD, we report that administration of defibrotide with same total daily dose employed for VOD in humans is also effective for both prophylaxis and treatment of murine aGvHD. This effect is mediated via a significant early inhibition of donor alloimmune effector T cell and neutrophil tissue infiltration, a substantial decrease in aGvHD‐associated tissue injury, and a reversal of plasma pro‐inflammatory/anti‐inflammatory cytokines ratios. These effects yield a significant increased survival. Mechanistic in vitro studies using human samples reveal that defibrotide inhibits peripheral blood leucocyte transendothelial migration by down‐regulating expression of key endothelial adhesion molecules involved in leucocyte trafficking such as E‐selectin, P‐selectin, ICAM‐1 and VCAM‐1. Collectively, these findings provide novel perspectives on the clinical applicability of this multi‐target drug in aGvHD therapy following allo‐HCT.

## 2 MATERIALS AND METHODS

### 2.1 Mice

BALB/c (H‐2K^d^, CD45.2) and C57BL/6J (H‐2K^b^, CD45.2) mice were purchased from Envigo Harlan (Huntingdon, Cambridgeshire, United Kingdom). At the time of allo‐HCT, mice were aged 10‐12 week. Housing was maintained under specific pathogen‐free conditions in the animal facilities of the University of Murcia. All animal protocols were approved by the Institutional Animal Care and Use Committee at University of Murcia (protocol A13150201).

### 2.2 Induction and assessment of murine aGvHD and histopathology analysis

A model of fully MHC‐mismatched allo‐HCT was carried out by transplanting bone marrow mononuclear cells from donor BALB/c mice into recipient C57BL/6J mice that were previously irradiated by with a lethal dose of 10 Gy split into two equal doses of 5 Gy and 24 hours apart (days −1 and +0). To induce aGvHD, on day +0 recipient C57BL/6J mice were injected intravenously with 1 × 10^7^ bone marrow mononuclear cells from donor BALB/c mice and 1.5 × 10^7^ BALB/c donor splenocytes. Mice survival post‐transplant was monitored daily, and clinical aGvHD was evaluated using a scoring system that generates a composite aGvHD score composed of individual scores for weight loss, posture, activity, skin integrity and fur texture.^19^

Histopathology analysis of aGvHD was performed in different sections of liver, intestine (colon), skin and oral mucosa (tongue) collected from the recipients 10 days after allo‐HCT and analysed as described previously^19^, ^20^ by a single pathologist blinded to the treatment groups. Samples from the different organs were fixed in formalin and embedded in paraffin. After, three‐micrometre sections were stained with haematoxylin and eosin stain for histopathologic examination. In brief, the skin histopathologic lesions were grade 0 (normal), grade I (slight vacuolar degeneration of epidermal basal cells), grade II (scattered individual apoptotic epidermal basal cells, spongiosis), grade III (separation of dermoepidermal junction) and grade IV (diffuse and severe ulceration, with extensive destruction of epidermis). Colon aGvHD scores were either grade 0 (normal), grade I (scattered individual apoptotic cells, with inflammatory cell infiltrate), grade II (crypt epithelial cell apoptosis, with villous blunting and/or exploding crypts), grade III (focal mucosal ulceration, with moderate villous atrophy) and grade IV (diffuse and severe mucosal ulceration). Liver aGvHD was scored according to degree of bile ducts affected: 0 (normal), grade I (≤25% bile ducts affected), grade II (25%‐49% bile ducts affected), grade III (50%‐74% bile ducts affected) and grade IV (≥75% bile ducts affected). The scoring system used for histopathologic evaluation of the tongue samples was based on intraepithelial inflammatory infiltrate as follows: grade 0 (absence of inflammatory infiltrate), grade 1 (weak), grade 2 (moderate), grade 3 (intense) and grade 4 (severe). The degree of T‐lymphocyte infiltrate or induction of apoptosis was evaluated by immunohistochemical labelling by using a commercial kit (EnVision Flex, Dako‐Agilent Technologies, Barcelona, Spain). Briefly, tissue sections were submitted to deparaffinization and rehydration, followed by antigen demasking procedure and endogenous peroxidase inhibition. After, sections were incubated with primary antibodies (anti–T‐CD3, Dako) for 1 hour at 37ºC, following by secondary anti‐rabbit labelled polymer incubation for 20 minutes at 37ºC. Immunoreaction was finally revealed by incubation with 3‐3´diaminobenzidine (Sigma‐Aldrich, St. Louis, MO). Positive immunoreaction was identified as a dark‐brown pericellular staining. In addition, polymorphonuclear (PMN) neutrophils were identified on H&E‐stained sections according to morphologically evident segmented nuclei. All analyses were performed by using a standard direct‐light microscope (Axio A10, Carl Zeiss, Barcelona, Spain).

### 2.3 Defibrotide administration

Defibrotide solution was provided by Gentium SpA, Jazz Pharmaceuticals (Dublin, Republic of Ireland). Defibrotide was suspended in saline solution at a concentration of 2,5 µg/µL to reach 25 mg/kg bodyweight, and 200‐250 µL was injected intraperitoneally (i/p) as a single dose as prophylaxis (day −2 to day +17 post‐allo‐HCT) or treatment (day +7 to day +27 post‐allo‐HCT).

Untreated controls were injected daily with same volume of saline solution without defibrotide.

### 2.4 Determination of plasmatic cytokines by enzyme‐linked immunosorbent assays (ELISA)

Murine IFNγ, TNFα, IL‐6, IL‐12, TGFβ and IL‐10 were measured in blood plasma by ELISA (Diaclone, bioNova Cientifica, Madrid, Spain; and Elabscience, Bethesda, MD). All experimental samples and standards were assayed in triplicate according to the manufacturer's instructions.

### 2.5 Cell cultures

SK‐Hep1, an immortalized human endothelial cell line derived from ascitic fluid, and primary human umbilical vein endothelial cells (HUVEC) were used to investigate defibrotide effects on endothelial adhesion molecule expression and human leucocyte adhesion/transendothelial migration in vitro. SK‐Hep1 was cultured in Eagle's Minimum Essential Medium (Sigma‐Aldrich) containing 10% foetal bovine serum (Gibco, Carlsbad, CA), 1% L‐glutamine (Gibco), 100 U/mL penicillin and 100 µg/mL streptomycin (Gibco) at 37ºC and 5% CO2. Cells were used between the tenth and fifteenth passages.

HUVEC were isolated from human umbilical veins and cultured in Endothelial Cell Growth Medium (PromoCell, Heidelberg, Germany) at 37ºC and 5% CO2 according to the manufacturer's instructions. Cells were used between the second and third passages.

Human peripheral blood mononuclear cells (MNCs) were isolated using Ficoll‐Paque density gradient centrifugation from heparinized blood samples obtained from healthy volunteers after informed consent. In some experiments, pooled sera samples obtained from three different patients with aGvHD on the day of diagnosis, or from same number of healthy donors, were used. Hospital Virgen de la Arrixaca and Hospital Clinic de Barcelona Ethics Committees approved the protocols used to obtain and process all human samples.

### 2.6 Parallel‐plate flow chamber adhesion assays

The effects of defibrotide on leucocyte adhesion on endothelial cells were analysed using a parallel‐plate perfusion chamber. SK‐Hep1 monolayers were treated previously with 100 µg/mL defibrotide for 24 hours or left untreated and then grown for 48 hours in medium supplemented with 20% pooled sera of aGvHD patients (n = 15, Table 1) or of healthy donors (n = 15). Then, SK‐Hep1 cells were perfused with control citrated blood from a healthy donor at a shear rate of 300 s^−1^ for 10 minutes. After perfusion, coverslips were washed with PBS and fixed in 3% paraformaldehyde in PBS. For leucocyte immunostaining, coverslips were treated with 1% BSA, permeabilized with 0.1% Triton‐X and stained with an anti‐human CD45 antibody (Abcam), followed by Alexa488‐conjugated anti‐rabbit secondary antibody (Life Technologies, Carlsbad, CA), and PE‐conjugated anti‐human CD3 (BD Biosciences, San Jose, CA) and DAPI (Sigma‐Aldrich) for nuclei counterstaining. Finally, coverslips were visualized by fluorescent microscopy (Leica‐DM4000B) through a video camera (Leica‐DFC310FX) and analysed using ImageJ software (National Institutes of Health, Bethesda, MD). The number of adherent leucocytes and T cells was expressed as percentage related to the total number of endothelial cells (number of leucocytes per 100 endothelial cells).

**Table 1:** Characteristics of acute GvHD patients and treatment

Patient Number	Birth Date	Diagnostic (Stage at Treatment)	Treatment	Date of transplantation	GvHD Prophylaxis	Donor
Sk‐HEP1 cells exposed during 48 h to a sera pool from three patients (P1, P2, P3) developing GvHD between day 7 and day 14. Sample from day 14.						
Patient 1	09‐01‐1971	Follicular Non‐Hodgkin's lymphoma	Cyclosporine A/Total Body Irradiation	15‐09‐2008	Cyclosporine A/Methotrexate	Non‐related
Patient 2	02‐01‐1980	Hodgkin's disease: nodular sclerosis	Fludarabine/Melphalan	21‐08‐2009	Cyclosporine A	Non‐related
Patient 3	23‐09‐1988	Non‐Hodgkin's lymphoma: Primary mediastinal large B cell	BEAM200 (BCNU‐Etoposide100‐Ara‐C‐Melphalan)	29‐04‐2009	Cyclosporine A/Methotrexate	Non‐related
Sk‐HEP1 cells exposed during 48 h to a sera pool from three patients (P4, P5, P6) developing GvHD between day 15 and day 21. Sample from day 21.						
Patient 4	05‐05‐1975	Acute myeloid leukaemia subtype M5 (AML‐M5)	Cyclosporine A/Total Body Irradiation	28‐08‐2009	Cyclosporine A/Methotrexate	Identical sibling
Patient 5	23‐09‐1988	Non‐Hodgkin's lymphoma: Primary mediastinal large B cell	BEAM200 (BCNU‐Etoposide100‐Ara‐C ‐ Melphalan)	29‐04‐2009	Cyclosporine A/Methotrexate	Non‐related
Patient 6	28‐02‐1983	Hodgkin's disease: nodular sclerosis	Fludarabine/Melphalan	18‐06‐2008	Cyclosporine A/Mycophenolate mofetil	Non‐related
Sk‐HEP1 cells exposed during 48 h to a sera pool from three patients (P7, P8, P9) developing GvHD between day 7 and day 14. Sample from day 14.						
Patient 7	19‐01‐1959	Biphenotypic Leukaemia (Relapse)	FLAG (Fludarabine‐ Ara‐C ‐G‐CSF)/IDA (idarubicin)/Melphalan	10‐03‐2009	Cyclosporine A/Mycophenolate mofetil	Non‐related
Patient 8	27‐04‐1957	Non‐Hodgkin's lymphoma: unclassified	BEAM200 (BCNU‐Etoposide100‐Ara‐C‐Melphalan	16‐12‐2009	Cyclosporine A/Mycophenolate mofetil	Non‐related
Patient 9	17‐03‐1995	Chronic lymphocytic leukaemia	BEAM200 (BCNU‐Etoposide100‐Ara‐C‐Melphalan	20‐07‐2009	Cyclosporine A/Mycophenolate mofetil	Non‐related
Sk‐HEP1 cells exposed during 48 h to a sera pool from three patients (P10, P11, P12) developing GvHD between day 14 and day 21. Sample from day 21.						
HUVEC cells exposed during 48 h to a sera pool from four patients (P10, P11, P12, P13) developing GvHD between day 7 and day 14. Sample from day 14.						
Patient 10	27‐03‐1968	Acute myeloid leukaemia subtype M5 (AML‐M5)	FLAG (Fludarabine‐Ara‐C ‐GCSF)/IDA (idarubicin)/Melphalan	31‐10‐2008	Cyclosporine A/Mycophenolate mofetil	Identical sibling
Patient 11	19‐12‐1948	Acute myeloid leukaemia subtype M1 (AML‐M1) (1st remission)	Fludarabine/Busulfan	22‐04‐2009	Cyclosporine A/Rapamycin	Identical sibling
Patient 12	03‐02‐1988	Acute myeloid leukaemia subtype M3 (AML‐M3)	Cyclosporine A/Total Body Irradiation	31‐10‐2008	Cyclosporine A/Methotrexate	Identical sibling
Patient 13	22‐04‐1958	Diffuse large B cell lymphoma	Fludarabine/Cyclosporine A/Melphalan/Total Body Irradiation	11‐01‐2019	Cyclosporine A/Tacrolimus/Mycophenolate mofetil	Identical sibling
Sk‐HEP1 cells exposed during 48 h to a sera pool from 3 patients (P13, P14, P15) developing GvHD between day 22 and day 28. Sample from day 28.						
Patient 14	20‐10‐1981	Hodgkin's disease: nodular sclerosis	Fludarabine/Melphalan	01‐12‐2009	Cyclosporine A/Rapamycin/Melphalan	Identical sibling
Patient 15	06‐01‐1968	Acute lymphoblastic leukaemia: T cell	Cyclosporine A/Total Body Irradiation	17‐07‐2008	Cyclosporine A/Methotrexate	Non‐related
Patient 16	14‐04‐1979	Multiple myeloma	Fludarabine/Melphalan	03‐03‐2009	Cyclosporine A/Mycophenolate mofetil	Non‐related

### 2.7 Transwell migration assays and flow cytometry analysis

HUVEC in culture were exposed to medium containing 20% pooled sera of aGvHD patients (n = 4, Table 1) or of healthy donors (n = 4) for 48 hours and incubated with 100 µg/mL defibrotide (treatment), or alternatively, by previous exposure to defibrotide for 24 hours followed by continuous incubation (100 µg/mL, added every 24 hours) before exposition to sera (prophylaxis). Afterwards, 7.5 × 10^4^ HUVEC were seeded onto the upper chamber of Transwell filters with 5‐µm pores (NUNC, Roskilde, Denmark). Then, 3 × 10^5^ peripheral blood MNCs or T‐CD3 cells (obtained by magnetic selection using Pan human T cell isolation kit (Miltenyi Biotec, Bergisch Gladbach, Germany)) were resuspended in 100 µL of DMEM medium (Sigma‐Aldrich) containing 0.3% bovine serum albumin (BSA) (Sigma‐Aldrich) and added to the upper chamber. The lower chamber contained DMEM medium supplemented with 0.3% BSA and 20% pooled sera of aGvHD patients/healthy donors as chemoattractant stimulus. Next, cells were allowed to migrate for 16 hours at 37ºC. Finally, the extent of cell migration was analysed using an automated cell counter (Bio‐Rad TC20, Hercules, CA). All experimental conditions were analysed in triplicate.

For flow cytometry analysis, HUVEC were detached with trypsin‐EDTA (Gibco), resuspended in PBS containing 1% BSA and incubated with fluorescence‐labelled anti‐human antibodies specific for E‐selectin, P‐selectin, VCAM‐1 and ICAM‐1 (all from eBioscience, San Diego, CA), or their control isotype antibodies for 30 minutes in the dark at 4ºC. Finally, cells were washed twice and acquired in a FACSCanto flow cytometer (BD Biosciences, San Jose, CA).

### 2.8 Statistical analysis

Results are expressed as mean ± standard deviation. Student's paired *t* test or one‐way ANOVA followed by Bonferroni's post hoc comparison tests was used for statistical comparisons between groups. Survival curves were plotted using Kaplan‐Meier estimates and statistically analysed using the Mantel‐Cox log‐rank test. *P* values < .05 were considered statistically significant.

## 3 RESULTS

### 3.1 Defibrotide aGVHD treatment reduces aGvHD clinical manifestations and reduces level of donor T cell and PMN neutrophil infiltrates in aGvHD‐target organs

**Figure 1:**
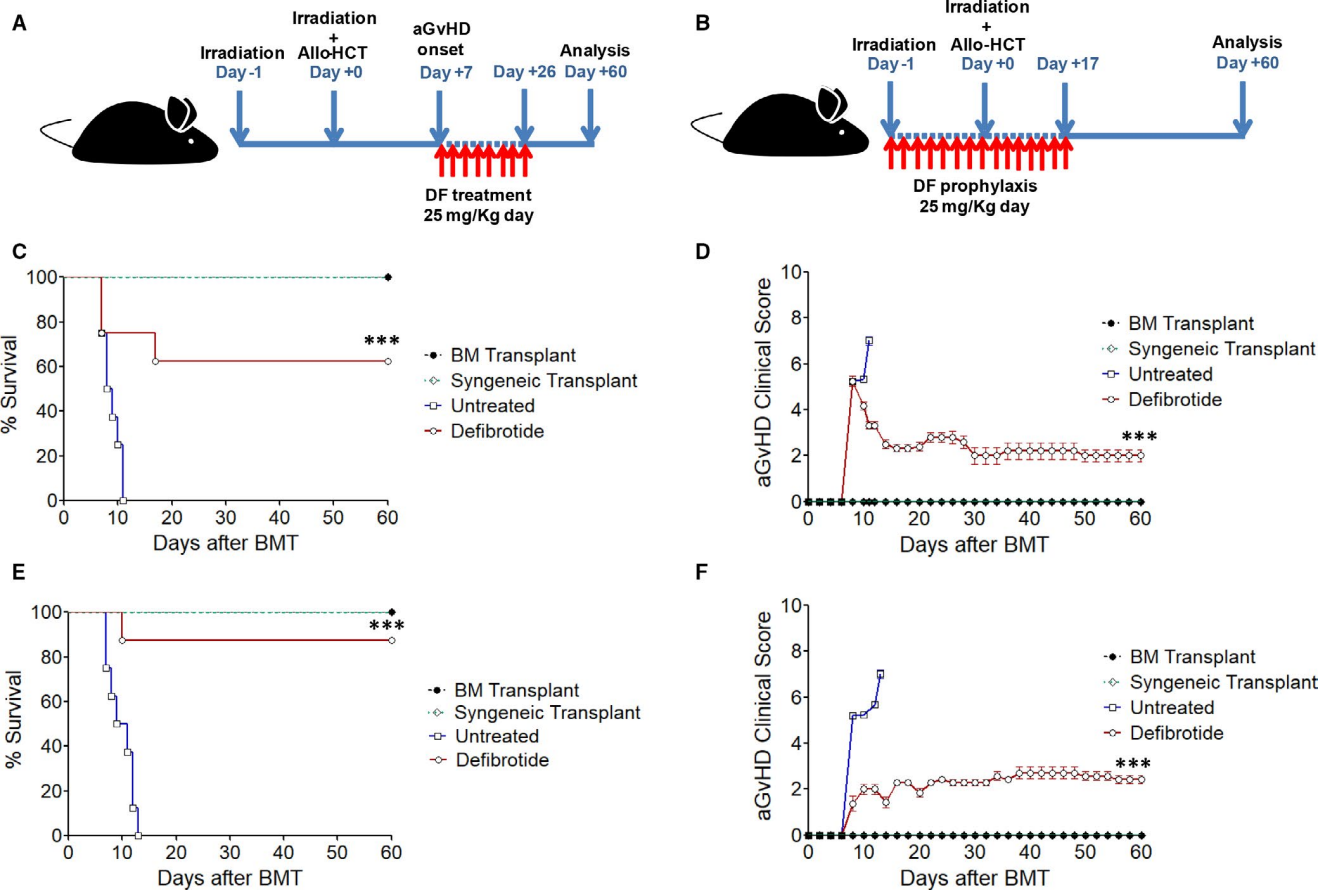
Defibrotide treatment or prophylaxis reduces the incidence and severity of aGvHD in mice undergoing a fully MHC‐mismatched allo‐HCT. After aGvHD onset (from day +7 after allo‐HCT), mice were treated with a daily administration of 25 mg/kg of defibrotide for 20 d or with 25 mg/kg of prophylactic defibrotide from 2 d before allo‐HCT to 17 days after. Schematic diagrams of the experimental protocols of defibrotide treatment (A) or prophylaxis (B) after allo‐HCT are shown. Untreated mice received same doses and volume of saline solution. C,D, Kaplan‐Meier survival curves and aGvHD clinical score of recipient C57BL/6J treated with defibrotide after aGvHD onset or (E,F) receiving prophylactic defibrotide are shown, respectively. Survival or aGvHD clinical score was significantly higher compared to untreated group, ^***^ *P* < .001. Results are representative of n = 3 separate experiments, n = 8 mice per experimental group

To test the hypothesis that defibrotide has direct effects in aGvHD onset and/or evolution, mice with ongoing aGvHD (from day +7 after allo‐HCT) were treated with a daily i/p infusion of 25 mg/kg of defibrotide for 20 days, following the same total daily dose used in human VOD/SOS patients (schematic diagram shown in Figure 1A). Control mice receiving allo‐HCT (BM transplant, *that is* receiving donor BALB/c bone marrow cells w/o splenocytes) and mice receiving syngeneic transplant (*ie* transplanted with bone marrow cells and splenocytes from donor C57BL/6J) did not develop aGvHD, whereas mice receiving allo‐HCT plus donor BALB/c splenocytes without defibrotide treatment (*ie* ‘untreated’ group) showed a rapid severe aGvHD occurrence, which caused the early death in all mice (Figure 1C). However, daily administration of defibrotide showed a significantly lower aGvHD‐related mortality than untreated mice group (*P* < .001) (60% survival in defibrotide group versus 0% survival in untreated group at day +60 post‐transplantation, *P* < .001) (Figure 1C). Also, defibrotide‐treated mice significantly improved their clinical aGvHD score compared to untreated mice group, being significantly lower from the onset of the disease (*P* < .001) (Figure 1D). To evaluate whether defibrotide had effects in the prophylaxis of aGVHD, mice were treated with a daily intraperitoneal infusion of 25 mg/kg of defibrotide from 2 days before allo‐HCT to 17 days after (schematic diagram shown in Figure 1B). Remarkably, mice receiving daily prophylaxis with defibrotide displayed a high survival advantage over the untreated mice group (*P* < .001) (87.5% survival in defibrotide group versus 0% survival in untreated group at day +60 post‐transplantation) (Figure 1E), also showing a significant reduction in aGvHD clinical score during aGvHD evolution compared to untreated group of animals (*P* < .001) (Figure 1F). These in vivo results suggest that defibrotide can mitigate the severity of early‐stage aGvHD, both as the only prophylaxis or treatment drug.

**Figure 2:**
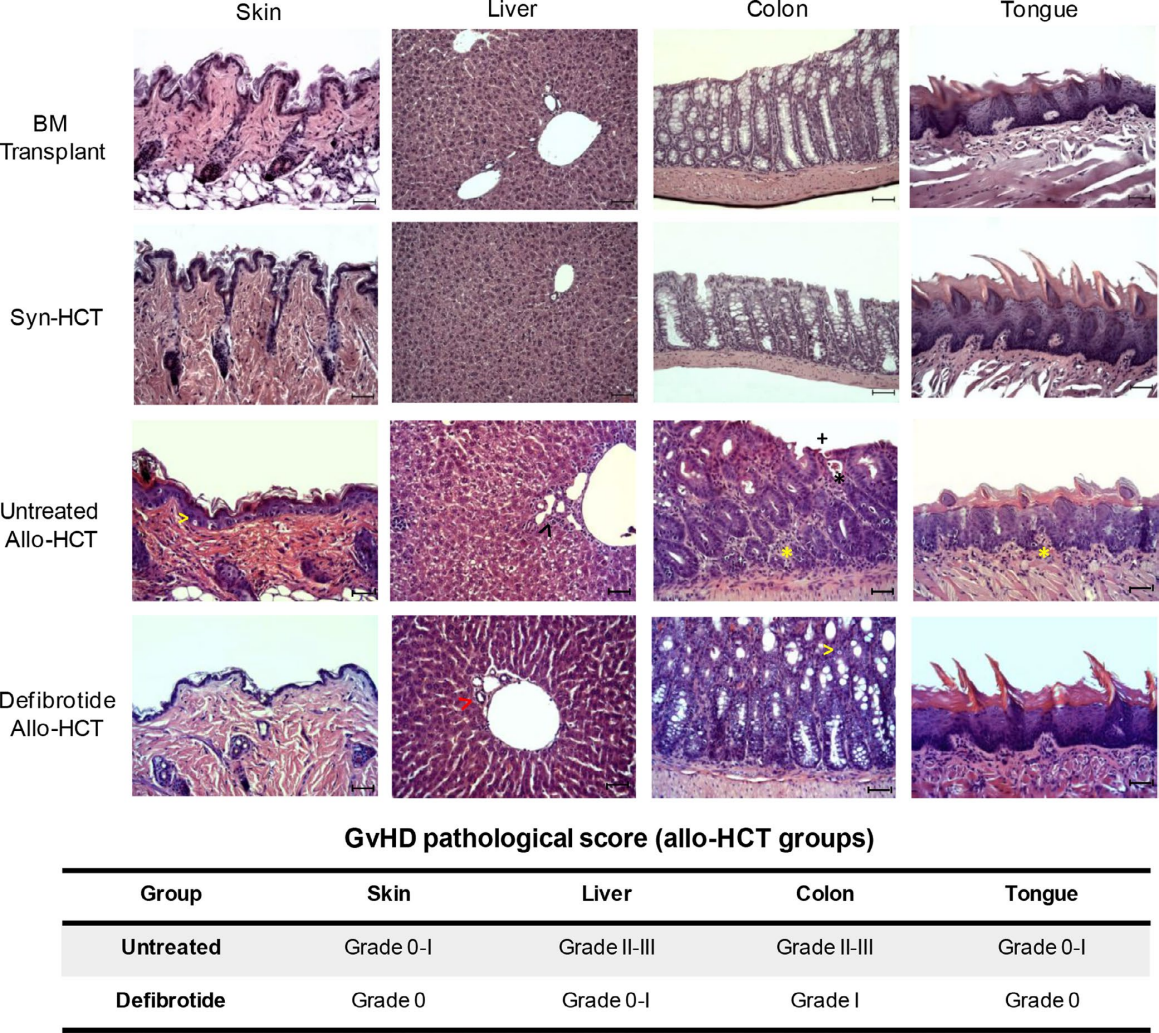
Histopathological assessment of aGvHD in mice receiving prophylactic defibrotide. Main histopathological aGvHD signs were analysed in skin, liver, colon and tongue of all groups of transplanted mice: BM transplant (w/o splenocytes), syngeneic transplant (syn‐HCT) and allo‐HCT mice, this last group including untreated mice or animals receiving prophylactic defibrotide at day +10 post‐transplantation. Skin: vacuolar degeneration and scattered apoptotic epidermal basal cells (yellow arrowhead); liver: 50%‐75% of bile ducts surrounded by inflammatory cell infiltrate and epithelial damage (black arrowhead), or >75% of unaffected bile ducts (red arrowhead); colon: moderate villous atrophy (+), interstitial inflammatory cell infiltrate (yellow asterisk), crypt epithelial cell apoptosis (black asterisk), or slight inflammatory infiltrate and scattered apoptotic cells (yellow arrowhead); and tongue: weak or moderate inflammatory cell infiltrate (yellow asterisk). H&E staining images from the different organs shown (magnification ×200) are representative of n = 8 animals per group. Scale bar: 50 µm

To determine the impact of defibrotide on reversal of aGvHD, a histopathology analysis was performed in the main aGvHD‐target organs (skin, liver, colon and tongue) in animals receiving prophylactic defibrotide infusions after allo‐HCT (Figure 2). On day +10 post‐transplantation, untreated mice displayed skin vacuolar degeneration and scattered apoptotic epidermal basal cells (grade 0‐I), whereas there were no patent lesions on the skin of the defibrotide‐treated, BM transplant or syngeneic (Syn)‐HCT animals. Histological examination of liver and colon displayed significant differences in the aGvHD severity between both experimental and control groups of animals. Whereas livers of untreated animals displayed an extensive epithelial damage, abundant periportal inflammatory infiltrate and destruction of the majority of bile ducts (grade II‐III), defibrotide‐treated group showed much lower aGvHD‐related hepatic epithelium damage (grade 0‐I). Also, colon of untreated mice showed moderate villous atrophy, interstitial inflammatory cell infiltrate and crypt epithelial cell apoptosis (grade II‐III), whereas those from animals receiving prophylactic defibrotide only displayed a slight inflammatory infiltrate and scattered apoptotic cells (grade I). In addition, the tongue was also analysed as a representative tissue of oral mucosa, which is also affected during aGvHD progression (Figure 2). Tongue dermis of untreated animals displayed a weak or moderate inflammatory infiltrate (grade 0‐I), which was absent in defibrotide‐treated mice. These observations suggest that the improved survival observed in the group of animals receiving defibrotide was correlated with a substantially reduced degree of tissue‐specific destruction.

**Figure 3:**
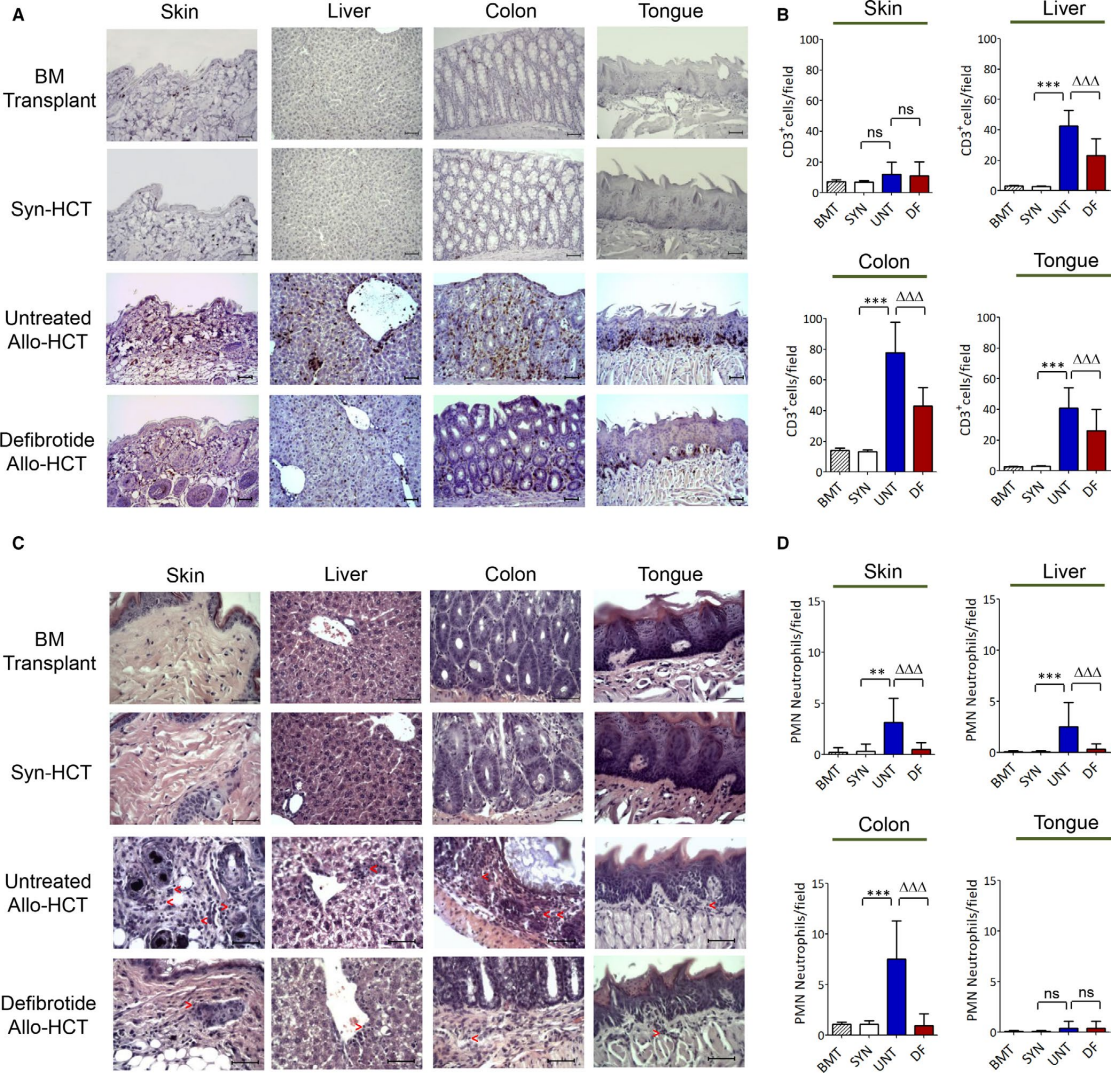
Defibrotide prophylactic administration significantly decreases donor T cell and PMN neutrophil infiltration in aGvHD‐target organs after allo‐HCT. Tissues from the different groups of animals, BM transplant (BMT), syn‐HCT (SYN) or allo‐HCT (either untreated (UNT) or receiving prophylactic defibrotide (DF)), were isolated 10 d after transplant. A, B, T cell infiltrates were detected by standard ABC anti‐CD3 immunohistochemistry procedure. C, D, Polymorphonuclear (PMN) neutrophils were identified in the different tissue sections on the basis to its morphological features (nuclear segmentation). Representative images of the skin, liver, colon and tongue from the different mice groups showing T‐CD3^+^ or PMN neutrophil infiltrates (red arrows) are shown (magnification ×200 or ×400, respectively) (n = 8 animals per group, n = 3 separate experiments). Scale bar: 50 µm. Absolute T‐CD3^+^ cell or PMN neutrophil counts are presented as mean ± SD per high‐power field from counts relative to 10 high‐power fields (magnification ×200 or ×400, respectively). T‐CD3^+^ cell or PMN neutrophil infiltrates were significantly increased compared to syn‐HCT group, **P < .01, ***P < .001, or decreased compared to untreated allo‐HCT group, ^∆∆∆^ *P* < .001, respectively

Tissue damage in aGvHD‐target organs is largely related with the ability of alloreactive donor T cells to traffic and infiltrate specifically these tissues.^21^, ^22^, ^23^ Thus, we next investigated levels of T‐CD3^+^ cell infiltrates in skin, liver, colon and tongue of untreated animals as compared to animals receiving prophylactic defibrotide at day +10 after allo‐HCT. Significantly, we observed that livers, colon and tongues of untreated allo‐HCT recipients displayed significant higher levels of T‐CD3+ infiltrates than BM transplanted or syn‐HCT animals. However allo‐HCT mice that received defibrotide showed a substantial decrease in T‐CD3^+^ infiltrates compared to their untreated counterparts, mainly in the colon (*P* < .001) (Figure 3A,B). On the other hand, skin sections of mice among the two groups of animals only displayed scattered infiltrates of donor T cells, which correlated with no observable lesions in this tissue at this time post‐transplantation.

Apart from tissue trafficking of T cells, it has been previously described that neutrophils can also infiltrate the aGvHD‐target organs, being considered as a bad prognostic marker of early allo‐HCT–associated mortality.^24^ As such, we also examined the level of neutrophil infiltrates in aGvHD‐target organs of all groups of animals. Although at day +10 post‐transplant mice remain still neutropenic, there were significantly reduced neutrophil infiltrates in skin, liver and colon of mice receiving prophylactic defibrotide compared to untreated counterparts after allo‐HCT, *P* < .001 (Figure 3C,D).

Importantly, control experiments revealed that peripheral blood lymphocytes and neutrophils counts in untreated mice and mice receiving defibrotide were parallel, indicating that the different degrees of tissue infiltration of both immune populations were not a consequence of a different leucocyte engraftment after allo‐HCT (data not shown).

### 3.2 Prophylactic administration of defibrotide reverses ratios of plasma pro‐inflammatory and anti‐inflammatory cytokines

**Figure 4:**
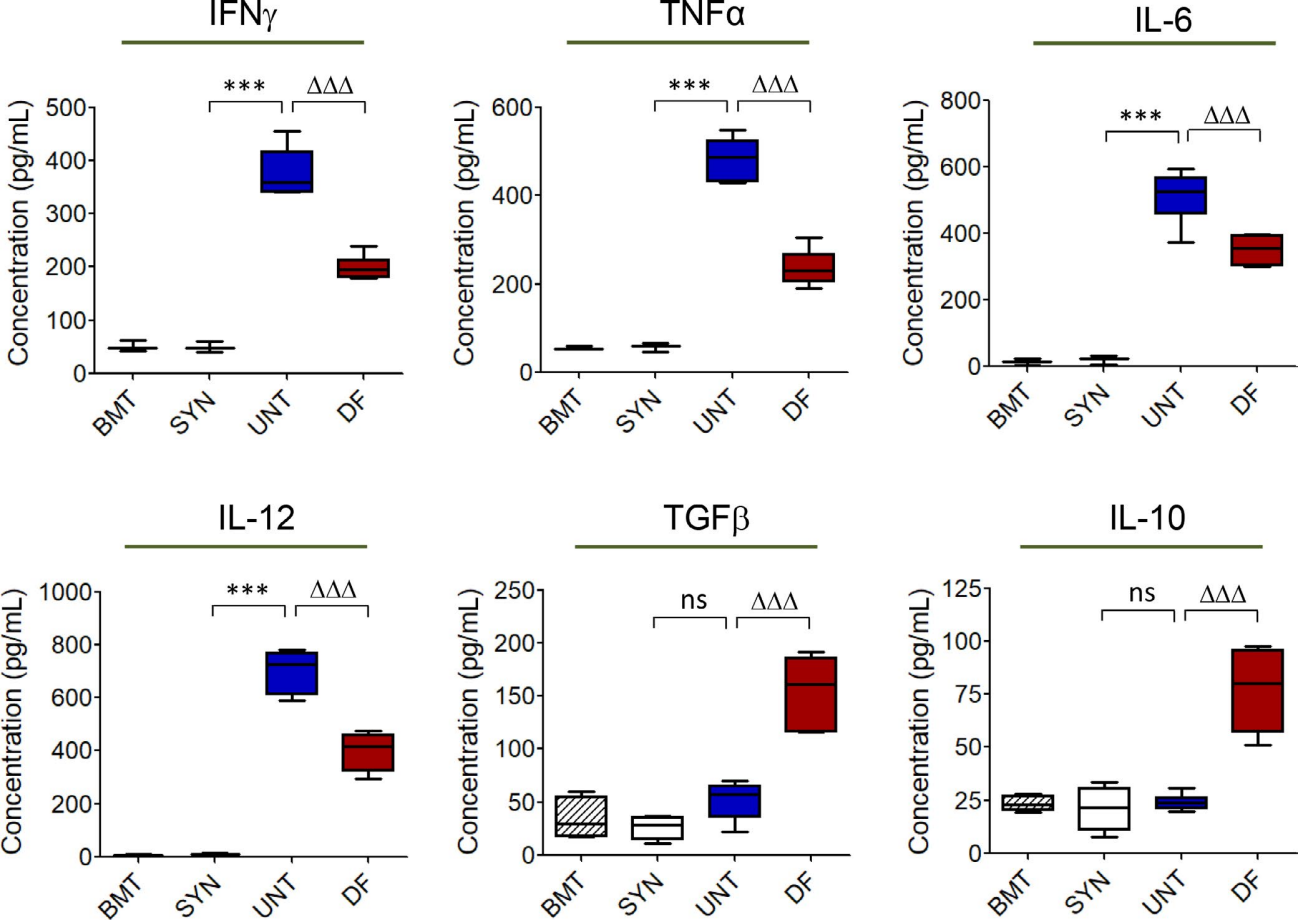
Defibrotide prophylaxis modifies the systemic profile of secreted plasma pro‐inflammatory and anti‐inflammatory cytokines on mice with ongoing aGvHD after allo‐HCT. Plasma concentrations of the pro‐inflammatory cytokines IFNγ, TNFα, IL‐6 and IL‐12, and anti‐inflammatory TGFβ and IL‐10 were determined on the different groups of animals, BM transplant (BMT), syn‐HCT (SYN) or allo‐HCT (either untreated (UNT) or receiving prophylactic defibrotide (DF)), on day +10 post‐transplant. Data are presented as mean ± SD of n = 5 animals per group and n = 3 separate experiments. Levels of each cytokine were significantly up‐regulated compared to SYN group, ^***^ *P* < .001, or significantly down‐ or up‐regulated compared to SYN group, ^∆∆∆^ *P* < .001, respectively

To further evaluate the effects of defibrotide administration on aGvHD development, we analysed the release of several pro‐inflammatory cytokines described to have key roles during the effector phase of aGVHD.^25^ Mice with ongoing aGvHD after allo‐HCT and without defibrotide administration (*ie* untreated animals) displayed significant increases in plasma pro‐inflammatory cytokines such as IFNγ, TNFα, IL‐6 and IL‐12 on day +10 post‐transplantation compared to syn‐HCT control group (w/o aGvHD) (*P* < .001), with simultaneous reduced concentrations of anti‐inflammatory TGFβ and IL‐10 (Figure 4). However, defibrotide prophylactic daily administration induced a marked decrease in these pro‐inflammatory mediators (*P* < .01, *P* < .001) and significant increased levels of the anti‐inflammatory cytokines TGFβ and IL‐10 (*P* < .01) (Figure 4).

### 3.3 Defibrotide impairs human leucocyte trafficking and down‐regulates endothelial adhesion molecules expression: mechanism of action involved in the protective effect of defibrotide.

**Figure 5:**
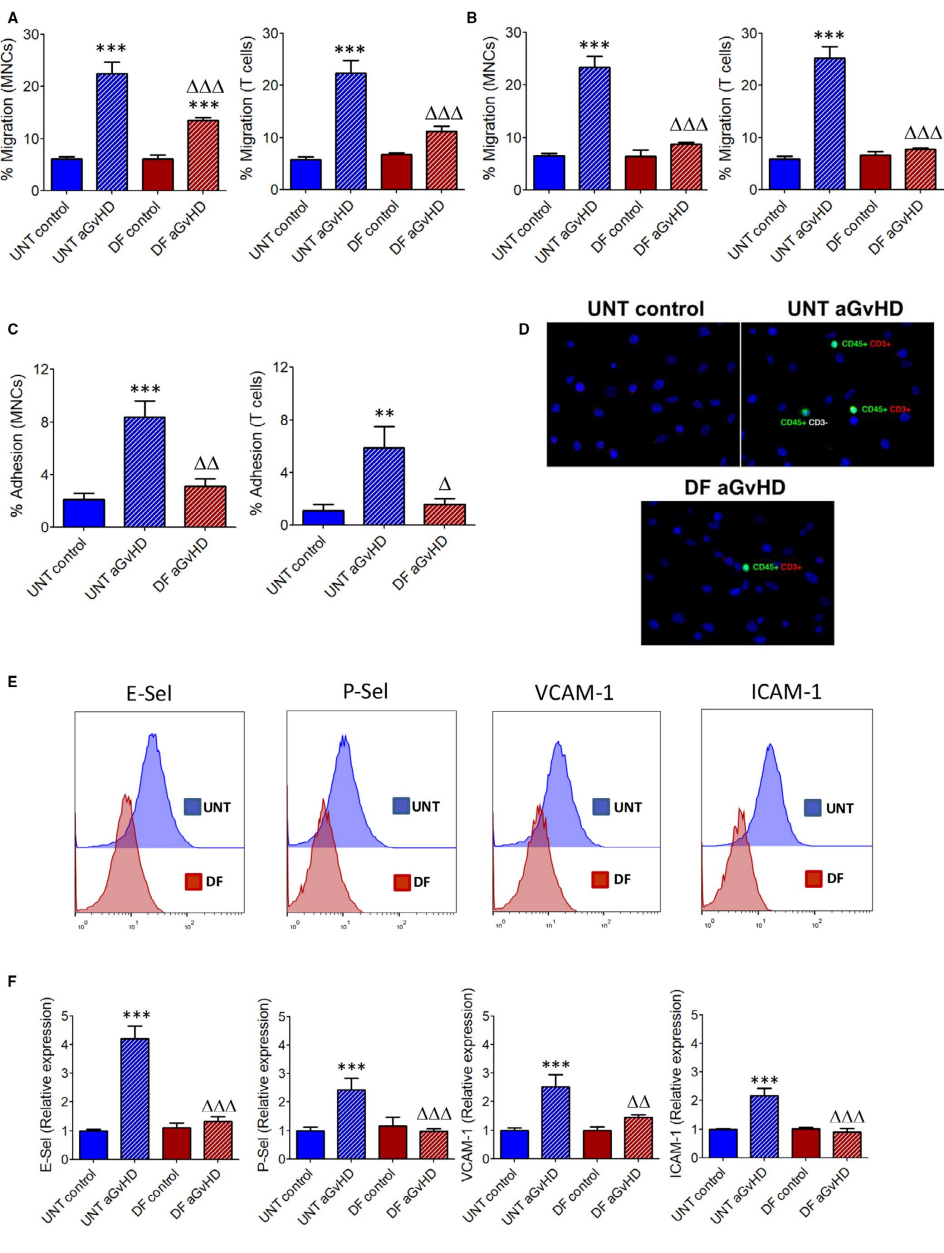
Defibrotide inhibits human peripheral blood MNC and T cell migration and down‐regulates endothelial adhesion molecules expression. A, HUVEC were exposed to culture medium containing pooled sera of aGvHD patients or from healthy donors (control) and treated with defibrotide, or (B) incubated with defibrotide before adding sera (prophylaxis). Then, peripheral blood MNC and T‐CD3 cell transendothelial migration towards aGvHD or control sera were analysed in Transwell assays in the presence or absence (‘untreated’, *ie* UNT) of defibrotide. Data are expressed as percentages of migrated cells related to the total number of MNCs or T‐CD3 cells added to the upper chamber. Migration was significantly increased in the aGvHD condition, ^***^ *P* < .001, or significantly inhibited after defibrotide incubation, ^∆∆∆^ *P* < .001, respectively. C, Also, effect of defibrotide on the MNC or T‐CD3 cell rolling and firm adhesion on SK‐Hep1 cells was analysed using a parallel‐plate perfusion chamber under dynamic flow conditions. Adhesion was significantly increased in the aGvHD condition, ^**^ *P* < .01, ^***^ *P* < .001, and significantly inhibited after defibrotide incubation, ^∆^ *P* < .05, ^∆∆^ *P* < .01, respectively. D, Representative micrographs showing adherent leucocytes (green staining with Alexa488‐conjugated anti‐human CD45, red staining with PE‐conjugated anti‐CD3 and blue staining with DAPI) obtained in the different experimental conditions are shown (magnification ×40). After, expression of endothelial adhesion molecules on HUVEC exposed to pooled aGvHD sera with DF or without prophylactic defibrotide (UNT) was analysed by flow cytometry. E, Representative histograms showing E‐selectin, P‐selectin, VCAM‐1 and ICAM‐1 expression on HUVEC in the different experimental conditions are shown. F, Relative expression of the different adhesion molecules was normalized to untreated HUVEC (*ie* w/o prophylactic defibrotide) incubated with healthy donor pooled sera (UNT control). Expression was up‐regulated in the aGvHD condition compared to UNT control, ^***^ *P* < .001, or down‐regulated compared to UNT aGvHD, ^∆∆^ *P* < .01, ^∆∆∆^ *P* < .001, respectively

Acute GvHD is associated with the up‐regulation of several endothelial cell adhesion molecules such as E‐selectin, P‐selectin, VCAM‐1 and ICAM‐1 in the affected organs in response to the effect of pro‐inflammatory cytokines, favouring donor alloreactive leucocytes to engage the endothelium and extravasate to the target tissue(s).^26^, ^27^, ^28^ In order to extrapolate the findings obtained in the aGvHD murine model to the human disease, we first analysed whether defibrotide interferes with peripheral blood MNC or T cell adhesion and migration on endothelial HUVEC and SK‐Hep1 cell lines. As previously reported,^11^ defibrotide does not interact with peripheral blood MNCs, and when leucocytes were directly incubated with defibrotide, leucocyte‐endothelial rolling adhesive interactions under hemodynamic flow conditions were not affected (data not shown). However, subsequent Transwell migration assays showed that when HUVEC were exposed to aGvHD sera and treated with defibrotide afterwards (Figure 5A) or before (Figure 5B), transendothelial migration of MNCs and T‐CD3 cells was significantly inhibited as compared to the levels of migration observed using untreated HUVEC (*P* < .001). Also, parallel‐plate perfusion chamber experiments showed that previous exposure of endothelial SK‐Hep1 cells to defibrotide significantly prevented MNCs and T‐CD3 rolling interactions and subsequent firm adhesion under hemodynamic flow conditions compared to untreated endothelial cells (*P* < .01 and *P* < .05, respectively) (Figure 5C,D).

The observed decreased interactions between leucocytes and endothelial cells suggest that defibrotide could modulate expression of key adhesion molecules involved in the sequential cascade of events that control migration of leucocytes from vascular to extravascular compartments. To this end, we analysed expression of E‐selectin, P‐selectin, VCAM‐1 and ICAM‐1 on HUVEC previously treated or not with defibrotide and after being stimulated with the aGvHD sera. Whereas untreated HUVEC displayed a significant marked up‐regulation of all these adhesion molecules (*P* < .001), previous incubation of HUVEC with defibrotide significantly down‐regulates expression of E‐selectin, P‐selectin, VCAM‐1 and ICAM‐1 (*P* < .01, *P* < .001) (Figure 5E,F). Collectively, these results indicate that defibrotide protective function in endothelium includes the down‐regulation of expression of endothelial adhesion molecules highly overexpressed during aGvHD, which could prevent the subsequent in vivo donor leucocyte extravasation to target tissue(s).

## 4 DISCUSSION

Allogeneic HCT provides a replacement of the diseased marrow with healthy hematopoietic progenitor cells derived from the donor. Moreover, allo‐HCT is an important type of adoptive cell immunotherapy in which donor alloreactive immune cells confer a potent graft‐versus‐malignancy effect. However, it can also induce immunopathological processes such as aGvHD, which is related to the immunologic recognition and destruction of specific host tissues that notably increase the mortality of the procedure.^29^

In the present study, we provide evidence on the therapeutic efficacy of defibrotide in prevention and treatment of aGvHD after allo‐HCT. Although two preliminary clinical studies have suggested indirect evidence that defibrotide prophylaxis might decrease the risk of aGvHD in paediatric and adult patients receiving allo‐HCT,^17^, ^18^ this is the first comprehensive preclinical study demonstrating that administration of defibrotide as a single agent, without other concomitant treatment, is sufficient to effectively prevent and/or treat this immunopathological complication.

Defibrotide has been previously described as a multi‐target drug, showing anti‐thrombotic/thrombolytic, anti‐ischaemic, pro‐fibrinolytic and anti‐angiogenic effects.^30^, ^31^, ^32^, ^33^, ^34^ But defibrotide has also demonstrated important anti‐inflammatory properties and a protective effect on endothelial cells from HCT conditioning.^12^, ^35^, ^36^ These findings and the fact that aGvHD occurrence is associated with higher activation and dysfunction of endothelial cells^37^ led us to hypothesize that defibrotide may be an effective agent for aGvHD prevention and/or treatment.

Our findings indicate that daily infusions of defibrotide employing the same total daily dose as in human VOD/SOS patients, given prior to allo‐HCT (prophylaxis) or once aGvHD has already developed (treatment), significantly dampened the severity of aGvHD, resulting in a much lower aGvHD clinical score and a markedly improved survival. Importantly, histological analysis demonstrates that infusions of defibrotide markedly restrain aGvHD‐target organ damage, particularly in the liver and colon compared to those that did not receive any treatment. Nevertheless, this allo‐HCT model did not allow evaluating the efficacy of defibrotide in reversing skin aGvHD. Skin manifestations are a frequent early complication during human evolving aGvHD.^38^ However, many studies point out that the choice of mouse strains to be used as donors and recipients of allo‐HCT greatly affects the development of the subsequent clinical manifestations of aGvHD due to differences in their immune triggering mechanisms (*ie* mismatches in major and minor histocompatibility complex molecules, effector T cell subpopulations involved or different production of pro‐inflammatory mediators).^39^, ^40^ Thus, these specific differences in spatio‐temporal GvHD‐target organ involvement depend largely on the murine model of HCT employed.

During the effector phase of aGvHD, donor effector T cells attack the recipient after recognizing the host tissues as antigenically foreign.^23^, ^41^ As expected, animals receiving allo‐HCT that developed aGvHD displayed high levels of T cell infiltrates in aGvHD‐target organs at early time post‐allo‐HCT (by day +10), mainly in liver and colon. However, mice receiving defibrotide prophylaxis before transplant displayed a marked and significant reduction of T cell infiltrates.

After T cell recruitment, aGvHD‐target organs are also infiltrated by other immunocompetent cells of the myeloid lineage, *that is* neutrophils, in a complementary innate pathway in which cognate T cell‐MHC interactions are not needed. Previous studies have revealed that an abundance neutrophil infiltrates in host tissues contribute synergistically to increase aGvHD severity and related mortality by production of reactive oxygen species that leads to T cell activation and subsequent potentiation of the tissue damage, mainly in the gastrointestinal tract.^24^, ^42^ In this study, we also found that similarly to what was observed with T cells, mice treated with prophylactic infusions of defibrotide showed markedly lower neutrophils infiltrates than allo‐HCT untreated mice.

The pathogenesis of aGvHD is closely linked to cytokines produced by T cells and other immune cells that infiltrate aGvHD‐target organs.^43^, ^44^ Here, we found that mice receiving prophylactic defibrotide displayed significant decreased systemic concentrations of a variety of pro‐inflammatory cytokines (*ie* IFNγ, TNFα, IL‐6 and IL‐12) as compared to allo‐HCT untreated animals, together with an increased production of anti‐inflammatory TGFβ and IL‐10. These data reveal that the administration of defibrotide prior to the aGvHD onset is sufficient to attenuate the systemic inflammation associated with aGvHD. This ‘shift’ in systemic cytokine levels from a pro‐inflammatory to an anti‐inflammatory profile has been described to have an important role in the improvement of the systemic inflammatory condition in several immune‐mediated pathologies, favouring the achievement of peripheral immunotolerance.^45^, ^46^ Moreover, modulation of systemic cytokines could down‐regulate the expression of endothelial adhesion molecules involved in the trafficking of alloreactive immune cells to the aGvHD‐target tissues.^47^, ^48^

Extravasation of circulating leucocytes across vascular wall involves a multistep adhesive interactions mediated by several molecules: a) selectins that mediate first tethering contacts and rolling; b) chemokines that mediate inside‐out integrin activation and c) integrins that mediate firm adhesion, crawling and subsequent transendothelial migration. It has been demonstrated in in vitro experiments and in vivo animal models that defibrotide decreases leucocyte extravasation by down‐regulating expression of endothelial P‐selectin or by interfering with the interaction between the integrin LFA‐1 and its endothelial ligand ICAM‐1.^49^, ^50^, ^51^, ^52^ Recent findings have also revealed that defibrotide suppresses the up‐regulated expression of VCAM‐1, ICAM‐1, VE‐cadherin and von Willebrand factor in endothelial cells exposed to sera from aGvHD patients.^53^ These reported findings, together with the observed results in our in vivo model of allo‐HCT and aGvHD, led us to hypothesize that the protective mechanism of action of defibrotide could be related to interruption of adhesive interactions between circulating lymphocytes and endothelium. With the purpose of extrapolating our in vivo results to what would occur in the human aGvHD condition, we studied whether defibrotide may affect cell‐surface expression of adhesion receptors on human endothelial cells, as well as its effects on human leucocyte adhesion and migration. Our results provide direct evidence that defibrotide down‐regulates the expression of critical endothelial molecules involved in leucocyte recruitment into tissues (*ie* P‐selectin, E‐selectin, VCAM‐1 and ICAM‐1) during aGvHD. This mechanism, together with the observed in vivo impairment in leucocyte adhesion and migration under the defibrotide effects, could underlie the observed improved clinical outcome of mice with aGvHD that received defibrotide.

In summary, our findings indicate that defibrotide, used as a concomitant prophylactic or therapeutic drug to the usual standard immunosuppressants employed for aGvHD, is effective in ameliorating the inflammatory response and tissue damage associated with this immunopathological disease, reducing its incidence and severity and significantly improving survival after allo‐HCT. In addition, defibrotide is not associated with relevant secondary side effects associated with other established treatments such as with corticosteroids. Therefore, defibrotide might represent a promising option for the prophylaxis and treatment of aGvHD in patients receiving allo‐HCT.

## CONFLICTS OF INTEREST

This research has been partially supported by Jazz Pharmaceuticals plc/Gentium Inc M.P and M.D‐R declare conflict of interest with Jazz Pharmaceuticals plc/Gentium Inc in the form of speaker's fee for symposia. E.C declares conflict of interest with Jazz Pharmaceuticals plc/Gentium Inc as consultant and in the form of speaker's fee for symposia. The remaining authors declare no competing commercial or financial interests.

## AUTHOR CONTRIBUTIONS

D.G‐B., JMM, RS, MB and E.C conceptualized and designed the study, and D.G‐B., MP, JEM‐R. and AIG‐G. performed all the experiments. CMM carried out and analysed all aGvHD histopathology experiments. D.G‐B., MP, CMM and RS analysed data. MB, RS, JMM, E.C, M.D‐R. and DGB developed the overall concept, designed and supervised all research, discussed the data and wrote the manuscript.

## Data Availability

The data that support the findings of this study are available from the corresponding authors upon reasonable request.
